# 3D imaging of magnetic domains in Nd_2_Fe_14_B using scanning hard X-ray nanotomography

**DOI:** 10.1107/S1600577524003217

**Published:** 2024-05-21

**Authors:** Srutarshi Banerjee, Doğa Gürsoy, Junjing Deng, Maik Kahnt, Matthew Kramer, Matthew Lynn, Daniel Haskel, Jörg Strempfer

**Affiliations:** a X-ray Science Division Argonne National Laboratory Lemont IL60439 USA; b MAX IV Laboratory Lund University 22100Lund Sweden; c Ames National Laboratory, Ames, IA50011, USA; Australian Synchrotron, Australia

**Keywords:** X-ray magnetic circular dichroism, XMCD, scanning transmission X-ray microscopy, STXM, tomographic reconstruction, imaging, Nd_2_Fe_14_B

## Abstract

Nanoscale structural and electronic heterogeneities are prevalent in condensed matter physics. Investigating these heterogeneities in 3D has become an important task for understanding material properties. To provide a tool to unravel the connection between nanoscale heterogeneity and macroscopic emergent properties in magnetic materials, scanning transmission X-ray microscopy (STXM) is combined with X-ray magnetic circular dichroism. A vector tomography algorithm has been developed to reconstruct the full 3D magnetic vector field without any prior noise assumptions or knowledge about the sample. Two tomographic scans around the vertical axis are acquired on single-crystalline Nd_2_Fe_14_B pillars tilted at two different angles, with 2D STXM projections recorded using a focused 120 nm X-ray beam with left and right circular polarization. Image alignment and iterative registration have been implemented based on the 2D STXM projections for the two tilts. Dichroic projections obtained from difference images are used for the tomographic reconstruction to obtain the 3D magnetization distribution at the nanoscale.

## Abstract 2

A computational imaging framework for visualizing 3D magnetization at the nanoscale has been developed.

## 1. Introduction

Magnetic materials play an important role in modern day industrial applications, from electric motors to sensors to high-density data storage and energy harvesting. The details of a material internal magnetization structure dictate several interesting properties. For example, electric motor cores are influenced by the presence of single domain walls and their displacements (Arrott, 2019 ▸). The internal magnetic structure is dictated by intrinsic properties such as lattice symmetry, spin–orbit coupling, exchange and dipolar interactions, as well as extrinsic properties such as structural defects acting as pinning centers, resulting in complex textures that affect macroscopic properties such as coercivity and remanence (Gutfleisch *et al.*, 2011 ▸).

In order to understand the behavior of magnetic systems, the determination of the internal magnetic domain structure is essential. In bulk magnets, probing these magnetic structures has been historically difficult, as they rely on indirect probing techniques. Tomographic techniques have been explored recently. In the first magnetic tomography experiments, neutron imaging was used to visualize internal magnetic fields (Kardjilov *et al.*, 2008 ▸) and magnetic domain walls (Manke *et al.*, 2010 ▸) in bulk magnetic systems with a spatial resolution of a few tens to hundreds of micrometres. Until recently, the investigation of smaller internal magnetic structures was limited to thin films and nanostructures. Photoemission electron microscopy (PEEM) as a surface-sensitive probe as well as X-ray magnetic circular dichroism (XMCD) in the soft X-ray range in transmission (Streubel *et al.*, 2015 ▸; Blanco-Roldán *et al.*, 2015 ▸) and Lorentz transmission electron microscopy (TEM) in combination with tomography (Phatak *et al.*, 2010 ▸, 2014 ▸; Tanigaki *et al.*, 2015 ▸) are widely used. Film thicknesses were mostly limited to not more than 200 nm in order to probe thin-film magnetism in transmission, allowing characterization of relatively thin 3D magnetic structures (Streubel *et al.*, 2016 ▸; Fernández-Pacheco *et al.*, 2017 ▸). The ability to probe actual 3D magnetic structures in the bulk of magnets at the nanoscale opens up a new dimension for exploration of a wide range of emerging properties and phenomena such as magneto-chiral effects (Hertel, 2013 ▸; Yan *et al.*, 2012 ▸), complex magnetic configurations (Donnelly *et al.*, 2017 ▸), high domain wall velocities (Hertel, 2016 ▸; Yan *et al.*, 2011 ▸) and symmetric spin-wave dispersion (Otálora *et al.*, 2016 ▸). The dynamics of nanoscale magnetic textures have been explored with soft X-ray PEEM and scanning transmission X-ray microscopy (STXM) using XMCD contrast (Schaffers *et al.*, 2019 ▸; Pile *et al.*, 2020 ▸). Full-field transmission tomography, proposed recently, offers significantly faster acquisition times for 3D magnetization mapping of thin films in the soft X-ray regime (Herguedas-Alonso *et al.*, 2023 ▸). In the last few years, hard X-ray dichroic ptychographic-tomography has been developed which offers high spatial resolution and large penetration depth, allowing the investigation of a number of extended magnetic systems (Donnelly *et al.*, 2017 ▸, 2018 ▸, 2020 ▸, 2022 ▸) and thus accessing the varying magnetic structures in the sample bulk of inherently 3D structures, including their dynamics (Finizio *et al.*, 2022 ▸). At the same time, hard X-ray dichroic tomography was used to obtain 2D magnetization maps by Suzuki *et al.* (2018 ▸), which then led to the visualization of skyrmions in 3D (Seki *et al.*, 2022 ▸) and the investigation of magnetization dynamics in Nd_2_Fe_14_B under applied magnetic fields (Takeuchi *et al.*, 2022 ▸).

In thick samples, reconstruction of 3D magnetic structures can be challenging and may require additional constraints. For instance, in electron tomography, the complexity can be reduced using the Maxwell equations (Phatak *et al.*, 2010 ▸, 2008 ▸). For experiments with soft X-rays, prior knowledge of the magnetic material has been incorporated (Streubel *et al.*, 2015 ▸, 2016 ▸). The angular dependence of the magnetic signal has also been exploited for retrieving the 3D magnetic vector field (Blanco-Roldán *et al.*, 2015 ▸). Although these experiments were carried out in the soft X-ray range, determination of 3D magnetization in the hard X-ray regime was pioneered by Donnelly *et al.* (2017 ▸) using 2D ptychographic projections. The increased X-ray absorption length for these photon energies enables acquisition of 2D projections for a large number of rotation angles and different sample tilts, which allow reconstruction of the 3D magnetic vector field by solving a set of simultaneous equations. By combining all tomographic projections, it is possible to recover the three magnetization components in a combined reconstruction (Donnelly *et al.*, 2018 ▸).

The main challenges in the development of X-ray based imaging techniques for the investigation of 3D magnetization in the sample bulk at the nanoscale are the following. First, limitations in the positional accuracy of the illuminated region during sample scanning causes misalignment of the 2D projections which leads to incorrect reconstruction of the final 3D magnetization structures of the material. Second, there are only a few appropriate tomographic reconstruction algorithms for recovering all three components of the magnetic vector field. Traditional tomography retrieves a scalar value at each voxel. However, for magnetic vector tomography, three components of magnetization need to be reconstructed for each voxel. Overall, computational imaging techniques for imaging 3D magnetization need further development since ptychographic methods for low-contrast magnetic imaging often suffer from reconstruction artifacts.

In this paper, we develop a computational imaging framework for visualizing 3D magnetization at the nanoscale. We present a combination of STXM with vector tomography to reconstruct magnetic domains in 3D, which is similar to the approach presented by Donnelly *et al.* (2018 ▸) using ptychographic projections. Compared with previous non-ptychographic hard X-ray dichroic tomography STXM studies (Suzuki *et al.*, 2018 ▸), which achieved 3D reconstructions of two components of magnetization density using one sample tilt in the measurements, the current method allows reconstruction of all three magnetization components of the magnetization density. Compared with ptychographic methods, the higher signal to noise ratio (SNR) of STXM due to integration over the total forward scattering intensity should be advantageous for magnetic imaging at resonances with lower dichroic contrast, such as the *K*-edges of 3*d* metals.

A novel coordinate descent based algorithm for alignment of the sample between the different orientations is introduced and the alignment of the object is an inherent iterative part of the magnetization reconstruction algorithm. The reconstruction pipeline can be applied to sets of 2D projections independent of how they are acquired. Compared with previous approaches (Donnelly *et al.*, 2017 ▸, 2018 ▸), the tomographic reconstruction algorithm for 3D magnetic domains presented here is implemented entirely in Python, eliminating the need to use commercial software, and enhancing transparency to ensure reproducibility of the results.

More specifically, we present an open-source end-to-end X-ray imaging algorithm to reconstruct the three components of the magnetization vector field in 3D for magnetic cylinders of a few-micrometres diameter with the entire imaging pipeline available on GitHub (Banerjee, 2023 ▸). This holds significance, especially given the subtle differences across different instruments, and can serve as a universal tool for users. The 2D projection images used in the workflow are obtained from STXM with circular dichroic contrast. The projections are aligned and registered based on a joint iterative reconstruction and reprojection method (Gürsoy *et al.*, 2017 ▸), which uses the principle of tomographic consistency. The magnetization is reconstructed in two perpendicular directions in 2D for the first tilt orientation of the sample. This is followed by a 3D rotation of the reconstructed object along with the magnetization to align it with the second tilt, and a subsequent reconstruction of the 3D magnetization. We demonstrate the applicability of our algorithm for two cylindrical pillars of single-crystal Nd_2_Fe_14_B, a material which forms the basis for the best permanent magnet currently available. We retrieve the 3D magnetization vector fields from the pillars with a resolution of close to 120 nm, which corresponds to the focal spot size of the X-ray beam used in the STXM experiment. Here, we explore bulk magnetic domains in single-crystal Nd_2_Fe_14_B which is unperturbed by defects or grain boundaries and allows a direct comparison with simulations.

## 2. Experimental setup

**Figure 1:**
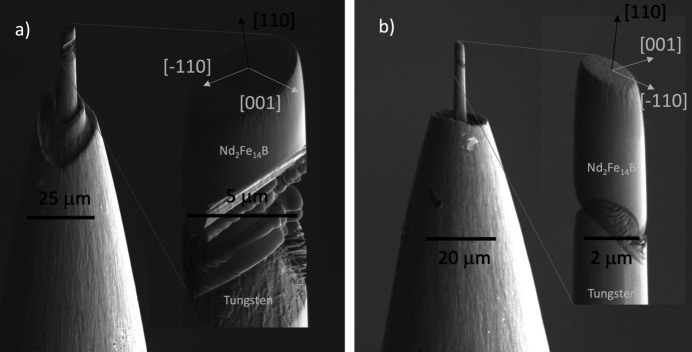
Electron microscope images of the (*a*) 5.4 µm and (*b*) 2.1 µm Nd_2_Fe_14_B single-crystal pillars mounted on a tungsten tip. The zoomed-in images show the approximate crystal orientation with the flat top facet normal aligned close to the [110] crystallographic axis. The 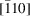 and [001] directions were inferred from experimental results, assuming moments are oriented predominantly along the [001] easy axis.

Two single-crystal Nd_2_Fe_14_B cylinders of 5.4 µm and 2.1 µm diameters were fabricated from flux-grown crystals at Ames National Laboratory (Wang *et al.*, 1998 ▸) using focused-ion-beam (FIB) milling. A single crystal of approximately 1 mm × 0.5 mm × 0.5 mm was aligned such that the long axis of the sample is the [110] direction, and [001] is orthogonal to the long axis. A rectilinear sample about 20 µm long and about 5 µm wide was extracted from the bulk crystal using Ga ion milling. The sample was then welded to a tungsten needle and further milled to form the cylindrical sample [Fig. 1 ▸(*a*)]. The procedure was repeated for the 2.1 µm sample [Fig. 1 ▸(*b*)]. The normal of the inclined, flat facets at the tip are closely aligned with the [110] crystallographic axis of the sample. The [001] direction, which lies close to the radial plane, is initially unknown but can be inferred from the magnetization direction within the individual magnetic domains since Nd_2_Fe_14_B is ferromagnetic and has an exceptionally high uniaxial magneto crystalline anisotropy along [001] at ambient temperature (Sagawa *et al.*, 1985 ▸).

**Figure 2:**
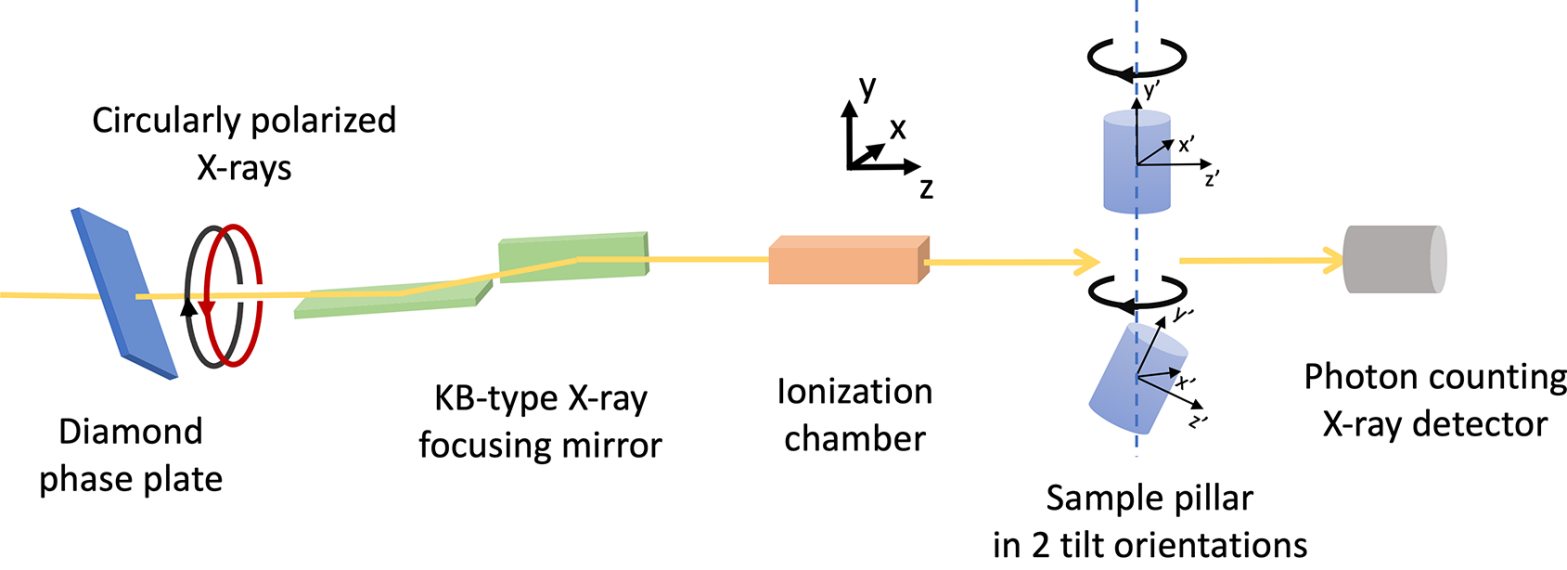
Schematic of the experimental setup for 3D magnetic tomography based on dichroic STXM with labeled laboratory and sample coordinate systems.

The STXM experiments were performed at the diffraction endstation (Carbone *et al.*, 2022 ▸) of the NanoMAX beamline (Johansson *et al.*, 2021 ▸) at the MAX IV Synchrotron, Lund, Sweden. A diamond phase plate with a 500 µm thickness was used to generate circularly polarized X-rays (see Fig. 2 ▸). The monochromatic circularly polarized X-ray beam was focused by Kirkpatrick–Baez (KB) mirrors, resulting in a focused beam of 120 nm × 120 nm full width at half-maximum (FWHM) at the sample position (Björling *et al.*, 2020 ▸; Kahnt *et al.*, 2022 ▸). The sample was mounted on a holder on top of a vertical rotation axis and a 2D scanner. Two different mounting holes in the sample holder allowed the sample to be mounted with the cylindrical axis along the vertical direction and at 30° from it. The transmitted X-rays were detected with an Eiger2 X 1M detector (Donath *et al.*, 2023 ▸) in order to simultaneously acquire the coherent diffraction patterns for ptychographic image reconstruction, which is not part of the work presented here. For the STXM signal the integrated intensity over the detector was used. The X-ray energy was tuned to 2 eV above the Nd *L*_2_-edge at 6727 eV in order to obtain maximum dichroic absorption contrast. 2D maps of the transmitted beam intensity were obtained using fly scanning vertically with an exposure time of 25 ms per point and an effective step size of 50 nm × 50 nm for the 2.1 µm pillar and 100 nm × 100 nm for the 5.4 µm pillar. Since the experimental setup was intended to acquire both STXM and ptychographic 2D projections, partial overlap between adjacent scan spots has been ensured from a ptychography point of view. However, in an experimental setup for only STXM, an overlap of the scanning positions is not required. Projections were acquired in an angular range of 360° with step size of 1° for incident left (*C*_L_) and right (*C*_R_) circularly polarized X-rays for both tilt angles while polarization was switched between *C*_L_ and *C*_R_ at each angular position. The 2D projections were used for the vector tomographic reconstruction of the 3D magnetic domains of the sample.

For magnetic contrast, XMCD is exploited, which has the maximum signal for the magnetic moment parallel to the X-ray propagation direction and zero when the magnetic moment is perpendicular (Siegmann & Stöhr, 2006 ▸). The XMCD signal has low magnitude with respect to the relatively noisy background of the acquired data, and hence the SNR is low. For each angular position, the integral of the magnetic component parallel to the X-ray beam is measured in a single projection. Thus, rotating the object around the vertical *y* axis perpendicular to the X-ray direction along *z* only probes the magnetization components 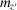 and 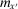 in the plane perpendicular to the rotation axis *y*. In order to reconstruct all three components of magnetization 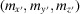, dual axis tomography is performed by recording the projection data in addition, with the sample tilted at 30° with respect to the rotation axis *y*, enabling access to the 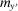 component as well. For a sample rotation axis perpendicular to the incident beam, a difference in tilt of 30° is sufficient to also reconstruct the third magnetization component in the Nd_2_Fe_14_B sample. Here we refer to the two tilts with 0 and 30° inclination as tilt-1 and tilt-2, respectively. The tilt angles can be any two non-identical angles of the sample axis tilted with respect to the tomographic rotation axis. However, in practice, this depends on the shape of the sample and a compromise has to be found between increased beam absorption and the reliability of measuring the vertical magnetization component. For similar cylindrical sample shapes, our choice of tilt angles was supported by prior studies in the literature (Donnelly *et al.*, 2018 ▸).

The XMCD contrast is defined as Δμ = μ^+^ − μ^−^, where μ^+^ = 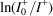 and μ^−^ = 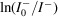 are the absorption contrasts determined from the incident (*I*_0_) and transmitted X-ray intensities (*I*) (Suzuki *et al.*, 2018 ▸). The + and − superscripts refer to *C*_R_ and *C*_L_X-ray polarization, respectively. The flipping ratio is defined by Δμ/(μ^+^ + μ^−^) and was found to be 1.1% at maximum contrast.

## 3. Alignment of projections

The stability of the experimental setup for acquiring projections is critical for the reconstruction of 3D magnetization structures at high resolution (*i.e.* limited by beam size only). For the 120 nm X-ray beam used in the experiment, a high positional accuracy and stability are desired. The stability may be compromised by vibrations and drifts in the experimental setup or instabilities in the X-ray beam, causing misalignment between the X-ray beam, sample and detector. Imperfections of rotation stage motion (runout error/spindle error) or drifts caused by small temperature variations can contribute to deterioration of resolution. Displacements of subsequent projections ultimately result in incorrect reconstruction of the 3D magnetization structures. There are various kinds of instabilities that can be attributed to fast fluctuations, slow movements or shifts. Fast fluctuations lead to blurring, while slow movements can lead to image distortions or image displacement. Fast fluctuations need to be avoided since they cannot be corrected for. On the other hand, slow movements can be corrected for using registration techniques.

In our case, the acquired projections are aligned with respect to a common tomographic rotation axis. For small or absent drifts, we can neglect image distortions and the object can be considered a rigid body, allowing rigid body registration using a two-step approach to compensate for shifts between acquisitions. Alignment of all projections is done for each polarization and tilt position separately followed by reconstruction of the 3D objects using the aligned projections. For each polarization, the 3D reconstructed scalar object from tilt-1 is rotated and aligned to the 3D reconstructed scalar object from tilt-2. Conversely, the reconstructed scalar 3D object from tilt-2 is aligned with the reconstructed scalar 3D object from tilt-1 after rotating it back. This is described in the following subsections.

### 3.1. Alignment of the tomographic axis

The imperfection of the rotation stage at small length scales, often called runout error, causes a noticeable shift of the rotation axis horizontally and vertically. However, all magnetic contrast projection images in the tomogram must have a common axis of rotation in order to allow an accurate 3D reconstruction of the object at high resolution. In the literature, techniques using fiducial markers (Cheng *et al.*, 2014 ▸; Han *et al.*, 2015 ▸), natural features such as corners (Brandt *et al.*, 2001 ▸), Canny edge detection (Duan *et al.*, 2008 ▸) or capacitance sensors to measure the runout errors of the rotation stage (Xu *et al.*, 2014 ▸) as well as tomographic consistency methods (Gürsoy *et al.*, 2017 ▸; Odstrčil *et al.*, 2019 ▸; Yu *et al.*, 2018 ▸) are used. Here, we use the joint reconstruction and reprojection (JRR) method (Gürsoy *et al.*, 2017 ▸), where the 3D object is reconstructed without considering sample jitter to obtain a low-resolution 3D volume. Subsequently, the reconstructed 3D object is projected back to the corresponding viewing angles through a reprojection process. The translation in the horizontal and vertical directions is corrected by registering the image pairs at different projection angles. Each image pair consists of the experimentally obtained projection image and the corresponding calculated reprojected image at the same projection angle. The cross-correlation between the image pairs provides us with the amount of horizontal and vertical shift to the experimentally acquired projection images to maximize this cross-correlation metric. Such shifts are obtained for all projections. Iterating over the reconstruction and reprojection steps *N* times improves the alignment of the projections and subsequently the resolution of the reconstructed 3D object.

The JRR algorithm is applied to all projections obtained with *C*_L_ as well as *C*_R_ polarization for one tilt. Projections corresponding to *C*_L_ and *C*_R_ for a tilt are concatenated in an array, along with the corresponding projection angles which result in a higher number of total projections and projection angles. Applying JRR alignment to these projections provides us with horizontal and vertical shifts for all projections and allows the alignment of projections corresponding to *C*_L_ and *C*_R_ only, as well as *C*_L_ and *C*_R_ with respect to each other. The intermediate tomographic 3D object reconstruction during the JRR alignment is done using the popular simultaneous iterative reconstruction technique (SIRT) (Gilbert, 1972 ▸; Herman & Lent, 1976*a* ▸,*b* ▸; Lakshminarayanan & Lent, 1979 ▸; van der Sluis & Van der Vorst, 1990 ▸). SIRT results in smoother reconstructions than other methods which allows for better alignment of the projections in the JRR algorithm. A similar JRR based alignment of the projections is repeated for the second tilt of the object.

### 3.2. Rotation of 3D object between tilts

**Figure 3:**
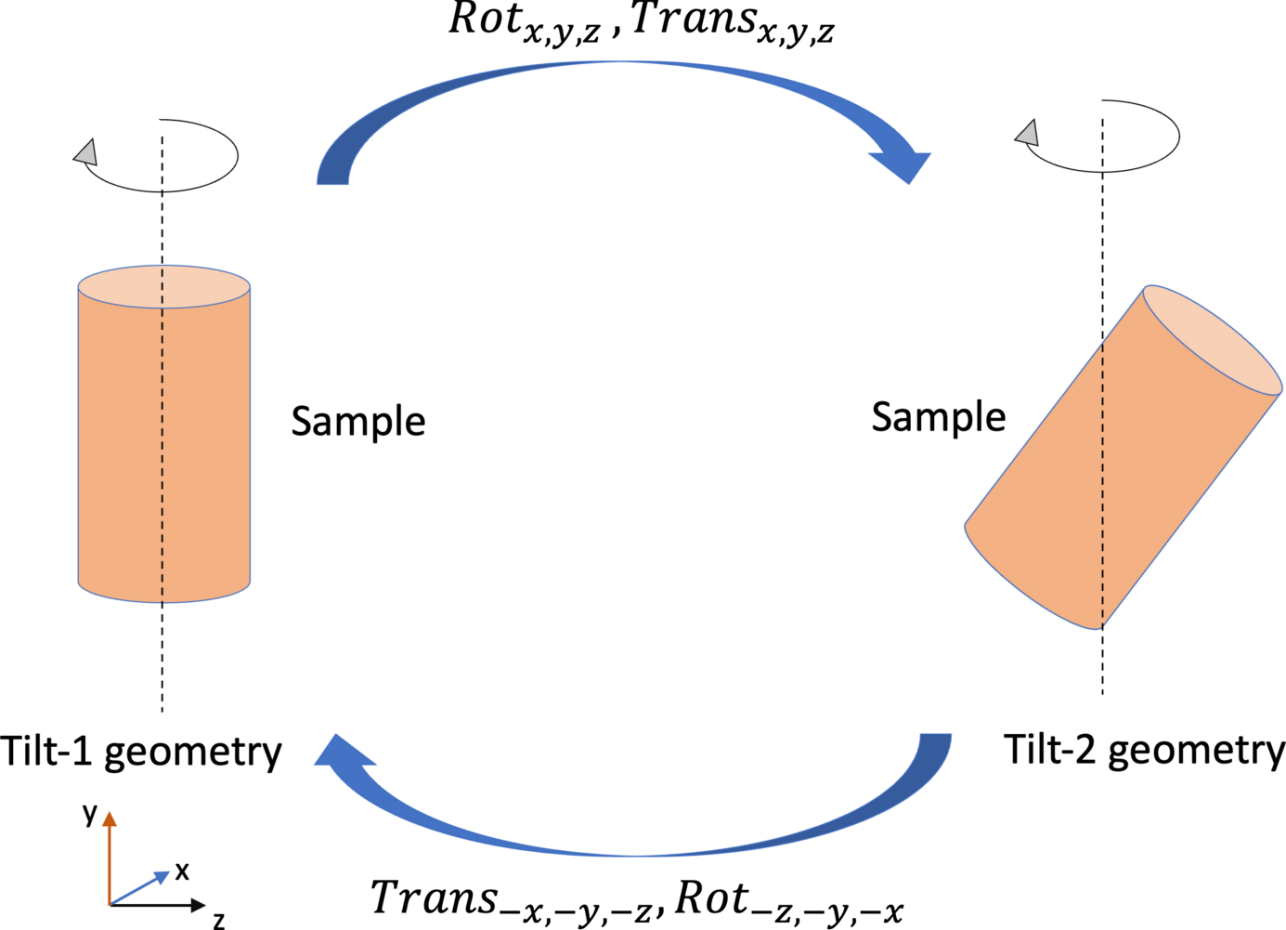
Rotation of the 3D reconstructed object between tilt-1 and tilt-2.

In order to perform a full 3D magnetization vector reconstruction, the geometric transformation between the orientations of the 3D objects reconstructed at the two tilt angles must be known (see Fig. 3 ▸). It is based on an initial guess of the transformation and subsequent further refinement using a conjugate gradient descent based algorithm which searches for the perfect alignment between the original 3D object in the tilt-1 orientation and the second 3D object rotated from tilt-2 into tilt-1 orientation. The aligned projections (see Section 3.1) corresponding to polarization states *C*_L_ or *C*_R_ are used to perform a scalar 3D reconstruction of the sample. The 3D object is reconstructed at tilt-1 and tilt-2 from the acquired projections using the *Gridrec* algorithm which is a Fourier grid reconstruction algorithm (Dowd *et al.*, 1999 ▸; Rivers, 2012 ▸). *Gridrec* provides us with sharper edges and boundaries of the 3D reconstructed object as it makes use of a gridding method for resampling the Fourier space from polar to Cartesian coordinates, offering both computational efficiency and negligible artifacts (Marone & Stampanoni, 2012 ▸). This enables accurate alignment of the 3D object at two different tilt orientations. Fig. 3 ▸ shows the rotation Rot_*xyz*_ around *x*, *y* and *z* in sequence followed by translation Trans_*xyz*_ along *x*, *y* and *z* in order to transform the 3D reconstructed object from the tilt-1 into the tilt-2 orientation. On the other hand, in order to re-orient the same 3D object from tilt-2 to tilt-1, the object must be translated by Trans_−*x*, −*y*, −*z*_ along *x*, *y* and *z* followed by a Rot_−*z*, −*y*, −*x*_ rotation around the *z*, *y* and *x* axes in sequence.

Based on the experimental setup for the Nd_2_Fe_14_B samples, we re-orient the 3D object from tilt-1 to tilt-2 by rotating the 3D reconstructed object around the *x* axis, followed by rotation around the *y* and *x* axes successively, followed by translations along *x*, *y* and *z*. In order to go back to tilt-1, we apply the reverse steps for the translation followed by rotations along the same axes sequentially with the same translation and rotation parameters.

Although the *Gridrec* reconstruction algorithm is used here for the alignment of the 3D object between the two tilts for efficient runtime, the alignment of the tomographic axes in Section 3.1 is done using the SIRT reconstruction algorithm for robustness to high-frequency noise. With SIRT, tomographic alignment is performed using cross-correlations to align between the original projections and the re-projections. The smoothness of the 3D reconstructed object aids in obtaining accurate alignment values. On the other hand, in order to obtain the 3D translation and rotation parameters between the 3D object in tilt-1 and transformed tilt-2 orientations, using *Gridrec*, the differential between the object in the two orientations is computed and used subsequently by a gradient descent algorithm to optimize the transformation parameters. This depends on the sharp reconstructed edges of the 3D object, which are emphasized by the *Gridrec* algorithm.

## 4. Reconstruction of 3D magnetization using vector tomography

**Figure 4:**
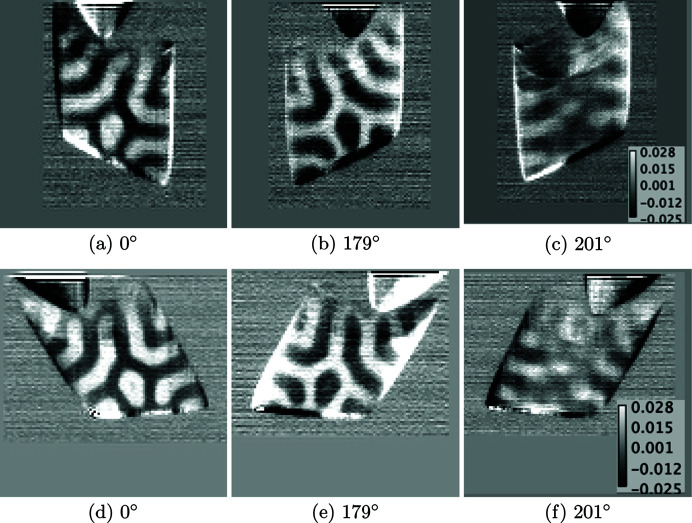
Projections corresponding to the XMCD absorption contrast (Δμ) of the cylindrical sample pillar for tilt-1 (top row) and tilt-2 (bottom row) for the same rotation angles. Panels (*b*) and (*e*) show the sample rotated by 179° with respect to (*a*) and (*d*), respectively. Panels (*c*) and (*f*) show the projection for rotation of the object by 201° with respect to (*a*) and (*d*), respectively. The pixel minimum and maximum values are shown in the bottom right corner of (*c*) and (*f*) as a bar chart.

After aligning the projections and the 3D reconstructed object corresponding to the two tilts as mentioned in Section 3, we reconstruct the 3D magnetic domains using vector tomography. We describe the magnetization using the local coordinate system (*x*′, *y*′, *z*′) for the 3D cylinder. The propagation direction of the circularly polarized X-rays is along the *z* axis with the tomographic rotation axis *y*, as shown in Fig. 2 ▸. The magnetic signal measured using XMCD is proportional to the magnetization component parallel to the X-ray beam along *z*. Thus, with the 3D object in tilt-1 orientation and the object being rotated around *y*, only the in-plane magnetization components 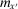 and 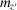 are captured in these projections. Some projections of magnetization contrast are shown in Fig. 4 ▸ (top row). On the other hand, with the 3D object in tilt-2 orientation and the object being rotated around the *y* axis, the contribution of the out-of-plane magnetization component 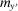 of the previous tilt angle is now measured along with the in-plane magnetization components 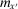 and 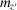. However, since the out-of-plane magnetization component for the present sample orientation is weak and the tilt axis happens to be along the hard magnetization axis, there is no visible change between the 2D STXM projections of the two tilts shown in Fig. 4 ▸. The 2D STXM projection data recorded by the detector correspond to the transmitted X-ray intensity after propagating through the Nd_2_Fe_14_B sample. Subsequently, the absorption coefficients μ^+^ and μ^−^ are computed for *C*_R_ and *C*_L_ polarized X-rays, respectively. Thereafter, μ^+^ and μ^−^ are used to compute absorption contrast Δμ. In Fig. 4 ▸, Δμ is plotted for both the tilt-1 and tilt-2 orientations.

**Figure 5:**
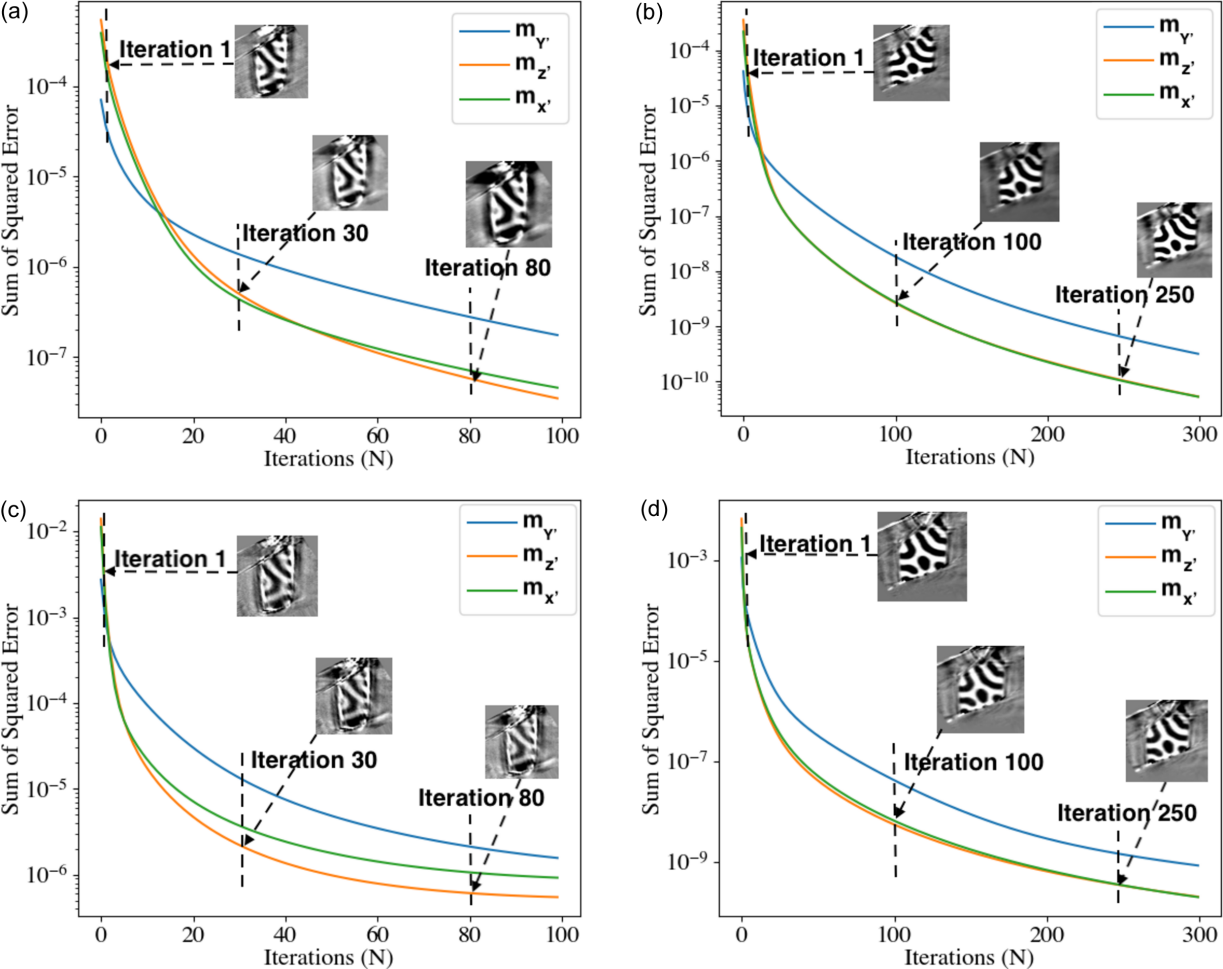
Sum of squared errors (SSE) over iterations (*N*). Left column: 2.1 µm-diameter sample; right column: 5.4 µm-diameter sample; top row: reconstruction done with one iteration for tilt-1 followed by one iteration for tilt-2; bottom row: reconstruction done with ten iterations for tilt-1 followed by ten iterations for tilt-2. The images correspond to a vertical slice in the middle of the sample for 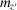.

The columns of Fig. 4 ▸ show the projections for the three projection angles 0, 179 and 201° for the same projected magnetic domains corresponding to tilt-1 (top row) and tilt-2 (bottom row). From these two tilt orientations of the 3D object, the entire magnetization 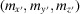 can be reconstructed using the SIRT algorithm, which shares similarities with the algebraic reconstruction technique (ART). SIRT employs a comparable updating strategy to ART but differs by employing all of the projections rather than a single one in updating the object (Phatak & Gürsoy, 2015 ▸). The SIRT algorithm is chosen for iterative reconstruction due to its fast convergence and it also does not assume any prior information based on noise or object model. Detailed equations for the 3D reconstruction of the magnetization have been explicitly covered by Hierro-Rodriguez *et al.* (2018 ▸). During the 3D vector magnetization reconstruction, the projections corresponding to tilt-1 are fed into the SIRT algorithm along with the initial 3D magnetization initially set to 0. After the magnetization vectors are reconstructed for tilt-1, they are rotated and subsequently fed into the SIRT algorithm as initial values for vector magnetization reconstruction along with the aligned projections corresponding to the tilt-2 orientation. Once the 3D magnetization vectors are reconstructed for tilt-2, they are rotated back to the tilt-1 orientation and again used as the initial value for reconstruction. This is done iteratively *N* times. *N* is determined by the fact that the relative change in the sum of squared errors (SSE) of the reconstructed 3D magnetic domains 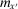, 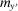 and 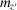 in the successive iterations is below a threshold of 4% per iteration. The decrease in log(SSE) over iterations follows an inverse power law. Before stopping this iterative algorithm, we observe that this criterion is satisfied for a few tens of iterations, in order to ensure its convergence properties over the iterations. The higher the number of iterations, the lower the SSE. In addition, for the 2.1 µm-diameter sample, fewer iterations were required for convergence compared with the 5.4 µm-diameter sample. The SSE in iteration *N* is given by the formula 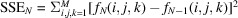, where *M* is the number of pixels along the *x*′, *y*′ and *z*′ directions in the 3D reconstructed volume. Fig. 5 ▸ shows the convergence of SSE between two successive iterations over a number of iterations (*N*). We show the reconstructed domains for both samples. For the 2.1 µm sample, the SSE converges faster than for the thicker 5.4 µm sample (see Fig. 5 ▸). Additionally, when one iteration is done for tilt-1 followed by one iteration for tilt-2 [see Figs. 5 ▸(*a*) and 5 ▸(*b*)], the convergence of the SSE is better compared with ten iterations for tilt-1 followed by ten iterations for tilt-2 [see Figs. 5 ▸(*c*) and 5 ▸(*d*)]. 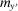 has the largest error among the reconstructed magnetic domains as the Nd_2_Fe_14_B sample is tilted by 30° from the vertical direction of rotation. Since the tilt angle of 30° is not large, the maximum contrast from 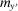 is reduced by a factor of two compared with 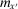 and 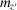.

**Figure 6:**
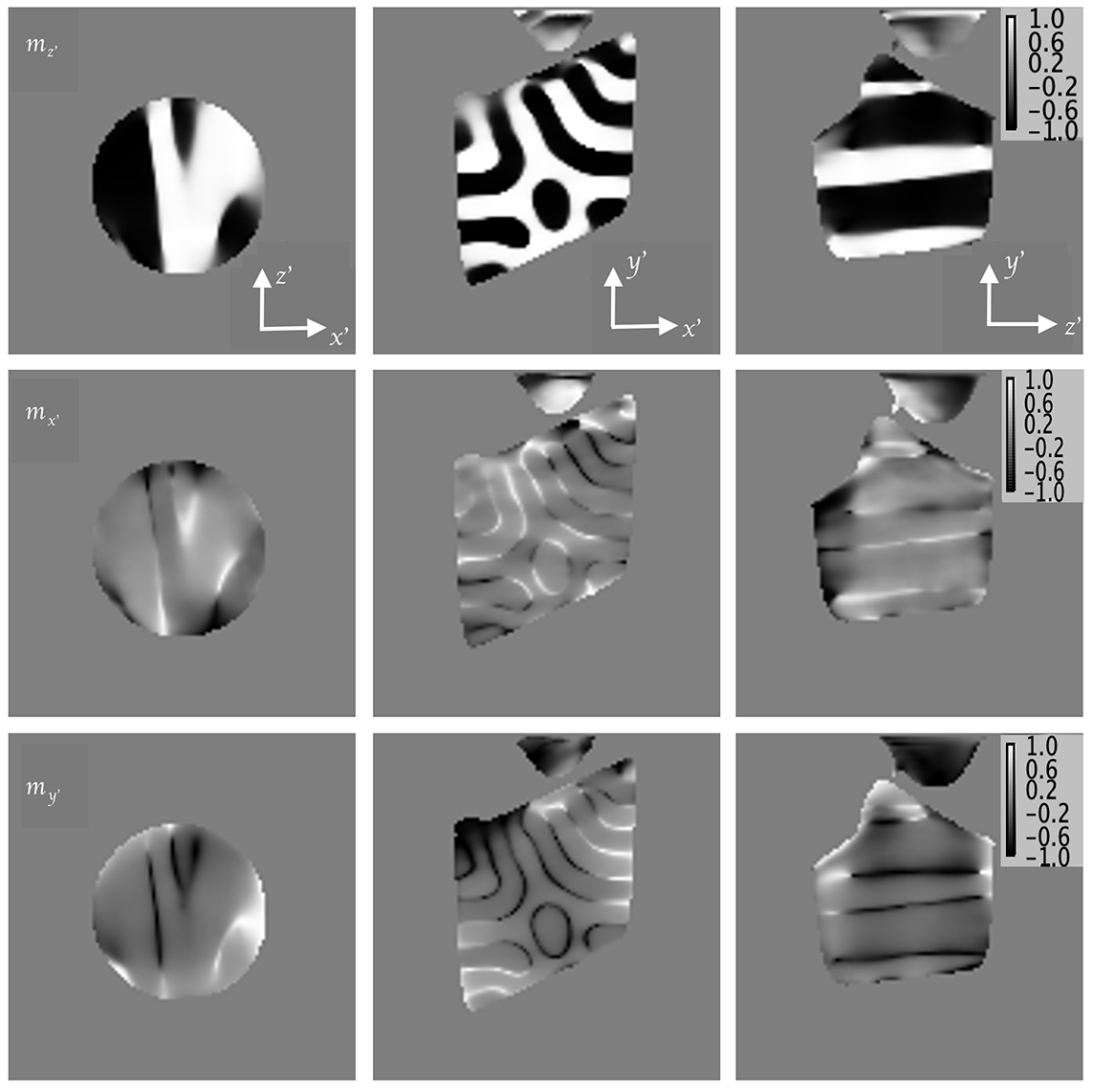
Horizontal (left column) and vertical (middle and right columns) slices through the center of the reconstructed 5.4 µm-diameter Nd_2_Fe_14_B sample. Top row: magnetization component 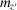 in the direction of maximum contrast. Middle row: magnetization component 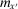. Bottom row: magnetization component 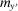. Left column: horizontal slice from the top. Middle and right columns: vertical slices of the sample from two perpendicular directions. The pixel minimum and maximum values are normalized to −1 and 1, respectively.

In the rest of the paper, for further analysis and visualizations, the 3D reconstructions after *N* iterations are utilized. Here, *N* corresponds to the iteration number where there is no significant change in log(SSE) over *N*. This corresponds to the 3D magnetization domain reconstructions at iteration 80 for the 2.1 µm-diameter sample and at iteration 250 for the 5.4 µm-diameter sample. At this iteration, the change in magnetization components 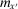, 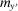 and 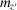 in the successive iterations is below the target threshold of 4% per iteration. In Fig. 6 ▸, orthogonal slices of the reconstructed 3D magnetization of the sample center are shown for components 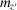, 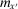 and 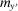. An additional scalar reconstruction of the sample in tilt-1 orientation, in which object pixels have higher values than the background, provides appropriate threshold to allow background removal. The surrounding background for the 3D shape of the reconstructed object was set to 0 accordingly. The local *z*′ axis of the Nd_2_Fe_14_B sample was defined as the in-plane direction with the largest magnetic contrast assumed to be aligned close to the crystallographic *c* axis (easy axis). Slices through the center of the 3D magnetization reconstruction for the 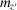, 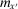 and 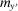 components are shown in the top, middle and bottom rows of Fig. 6 ▸, respectively, for the 5.4 µm-diameter sample. Contrast between adjacent domains for the other components 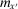 and 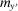 is low. Domain walls, although not actually well resolved, are clearly visible in the slices for 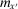 and 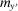. In order to confirm if the stripes within the object slices of 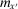 and 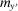 are indeed domain walls and not artifacts due to misalignment during object registration, a vector tomographic reconstruction was also done for the sum of the *C*_L_ and *C*_R_ projections for both orientations with the same algorithm. No stripes were observed in this case. This affirms the stripes observed in Fig. 6 ▸ are indeed domain walls although they are significantly broadened through the convolution with the probe size.

**Figure 7:**
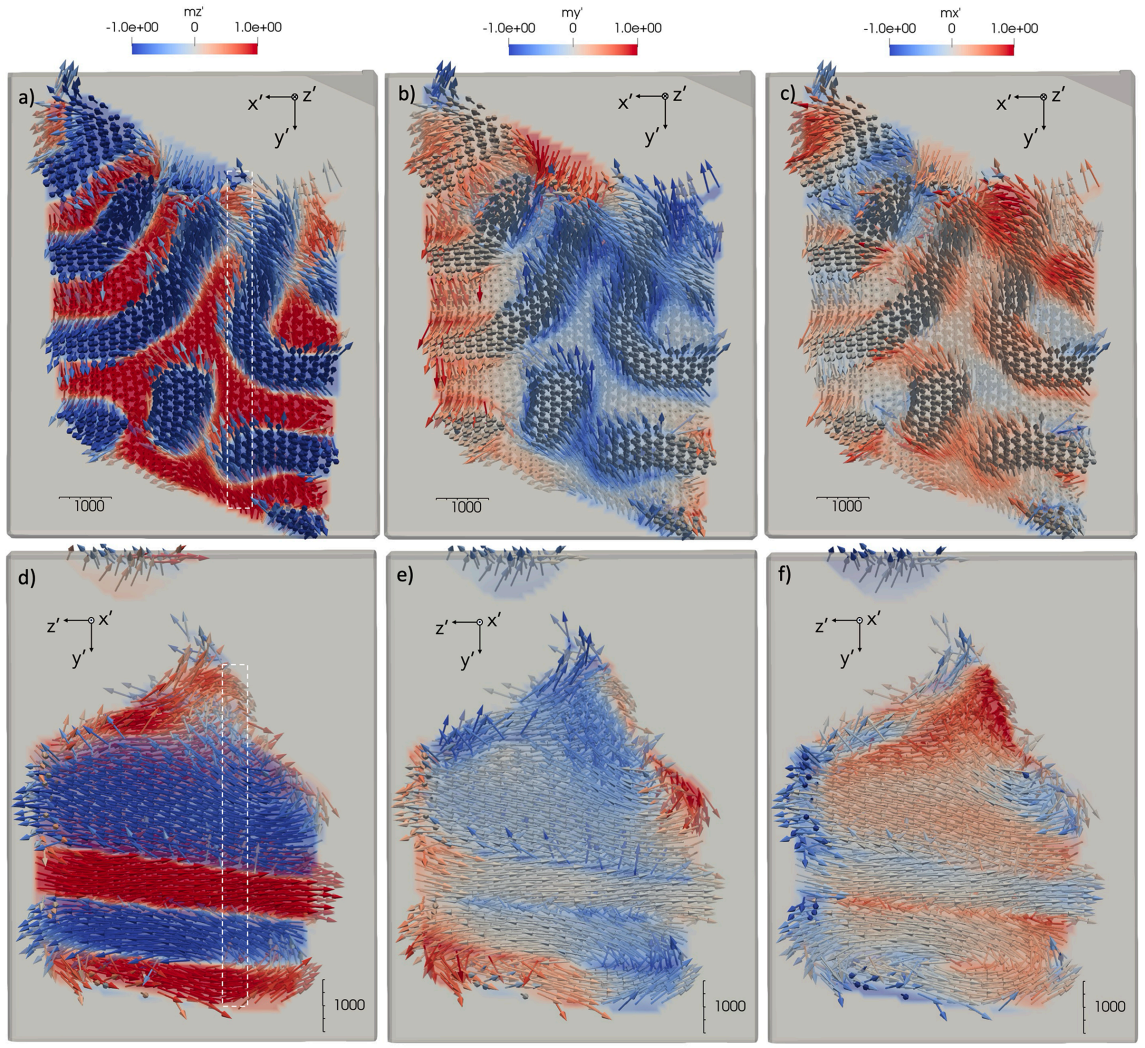
Slices of the reconstructed 5.4 µm-diameter sample in the orthogonal (*a*)–(*c*) *x*′*y*′ and (*d*)–(*f*) *y*′*z*′ planes with the moment directions visualized by vectors denoting the direction of the 3D magnetic moment. Colors denote moment components along (*a*) and (*d*) 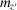, (*b*) and (*e*) 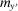, and (*c*) and (*f*) 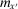, respectively, where 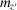 corresponds to the easy axis. The white rectangle in the *x*′*y*′ slice denotes the location of the *y*′*z*′ slice.

**Figure 8:**
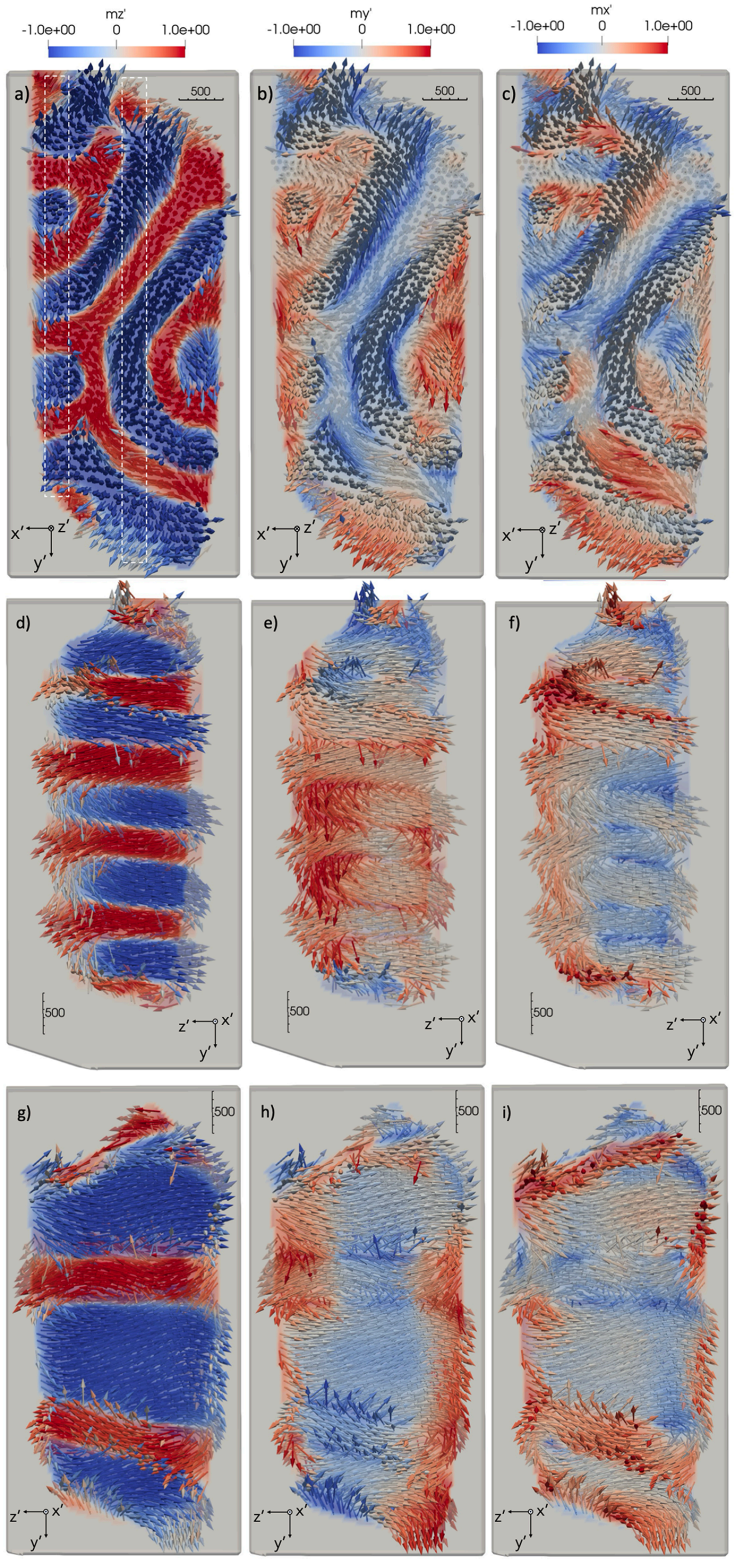
Slices of the reconstructed 2.1 µm-diameter sample in (*a*)–(*c*) one orthogonal *x*′*y*′; and (*d*)–(*i*) two *y*′*z*′ planes: (*d*)–(*f*) one close to the sample edge and (*g*)–(*i*) one in the sample center, with the moment directions visualized by vectors denoting the direction of the 3D magnetic moment. Colors denote moment components along (*a*), (*d*) and (*f*) 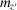; (*b*), (*e*) and (*g*) 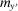; and (*c*), (*f*) and (*i*) 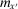, where 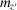 corresponds to the easy axis. White rectangles in the *x*′*y*’′ slice in (*a*) denote the locations of the *y*′*z*′ slices.

Visualization of the reconstructed 3D magnetization is carried out in *Paraview* in vector representation (Ahrens *et al.*, 2005 ▸; Ayachit, 2015 ▸) with the background removed. Magnetic domain vectors have been converted into 3D unit vectors. The background around the 3D object has been removed in order to visualize only the magnetic domains in the region of the 3D object of interest. The inner volume of the actual 3D object is visualized by 300 nm- (voxel size 60 nm) and 500 nm-thick (voxel size 100 nm) orthogonal slices for the 2.1 µm- and 5.4 µm-diameter sample, respectively. The reconstructed vector magnetization is shown in Fig. 7 ▸ for the 5.4 µm-diameter Nd_2_Fe_14_B sample. The magnetization within the domains points in anti-parallel directions mainly along the *z*′ axis. The 3D magnetization vectors are represented as unit vectors with the colors representing the respective magnetization along the *x*′, *y*′ and *z*′ directions. Color coding has been implemented for the 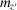, 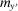 and 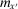 components in Figs. 7 ▸(*a*), 7 ▸(*b*) and 7 ▸(*c*), respectively, for the *x*′*y*′ slice; and 7 ▸(*d*), 7 ▸(*e*) and 7 ▸(*f*), respectively, for the *y*′*z*′ slice. A similar behavior is observed for the 2.1 µm-diameter sample in Figs. 8 ▸(*a*)–8(*i*). From Figs. 7 ▸(*d*) and 8 ▸(*d*), it can be seen that domains extend along the direction of the magnetic moments, which is assumed to be close to the easy axis [001], but vary arbitrarily within the *x*′*y*′ plane. This can be seen consistently through the bulk of the pillar. As can be seen from Figs. 7 ▸(*a*) and 7 ▸(*d*), domain walls are mostly orthogonal to the sample surface along the *x*′ and *y*′ directions, while oriented arbitrarily within the samples. The [110] direction points upwards close to the *y*′ direction. The 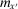 and especially the 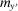 components visualize the rotation of the magnetic moments in the domain walls between the ferromagnetic domains as can be seen in Figs. 7 ▸(*b*) and 7 ▸(*c*). The domain size in the *x*′*y*′ plane of the 5.4 µm sample ranges from 600 nm to 800 nm in Fig. 7 ▸(*a*). Domains become smaller and the domain walls more extended in the 2.1 µm sample ranging from 300 nm to 400 nm in Fig. 8 ▸(*a*). Note the variations in size of the magnetic domains with a change in sample size, which has not been reported in the literature. However, we observe that the domain width to sample diameter ratio is comparable in both samples with 0.13 and 0.17 for the 5.4 µm and 2.1 µm samples, respectively, which is supported by micromagnetic simulations (Fullerton, 2024 ▸). The spatial resolution of the reconstructed 3D magnetization profile is close to the size of the focused beam of 120 nm FWHM in *x* and *y*, as estimated from the domain wall thickness in Fig. 8 ▸.

## 5. Discussion

The reconstruction of all three magnetization components of the magnetization density from STXM presented here profits from a favorably high SNR due to integration over the total forward scattering intensity, but spatial resolution is limited to a resolution equal to or worse than the beam size at the sample position. Although ptychography methods can reach higher spatial resolution, they are likely to be limited to hard X-ray absorption edges with rather large dichroic contrast of at least a few percent, while STXM methods may enable imaging at edges with lower dichroic contrast such as the *K*-edges of 3*d* metals.

Challenges during experiments arise due to imperfections in the sample tomographic rotation stage motion and random motion in the alignment between the X-ray beam, sample and detector. This becomes prominent for imaging of magnetization domain features at a spatial resolution on the order of hundreds of nanometres. Moreover, STXM data are acquired for *C*_L_ and *C*_R_ polarized X-rays for two tilt orientations with the rotation stage stability becoming crucial for the entire duration of the experiment.

The total data acquisition time for one sample was 3 d for the two polarization states at both tilt orientations. The stability of the experimental setup over this time is near impossible to maintain and needs to be corrected. We considered rigid body motion between the STXM projections and aligned the projections accordingly. However, using non-rigid body motion for alignment correction would be of interest in future work to account for drifts within individual projections. The use of interferometry for registration of sample and beam position to aid in alignment is also being explored. A setup with the tomographic rotation axis inclined with respect to the incident beam, as in a laminography setup, would offer the advantage of being able to perform the experiment for just one tilt angle. This would require less acquisition time and no 3D alignment of the sample between the tilt angles. However, the reconstruction of the magnetic domains from such a setup is expected to pose challenges and further disadvantages like a missing cone (Holler *et al.*, 2019 ▸) and has not been attempted here.

The reconstruction algorithm uses a CPU based implementation of *Tomopy* (Gürsoy *et al.*, 2014 ▸; Hierro-Rodriguez *et al.*, 2018 ▸) and each iteration corresponding to one reconstruction from tilt-1 and tilt-2 data takes approximately 1 min on a CPU. In order to speed up the reconstructions, a GPU based implementation is desirable. A workflow can be built for experimentally acquiring the STXM projections, registering these projections based on tomographic consistency or other methods, and finally performing the 3D vector magnetization domain reconstruction in *Tomopy*. The algorithm developed here is released as an open-source software on GitHub (Banerjee, 2023 ▸).

With construction of the POLAR beamline as part of the Advanced Photon Source Upgrade (APS-U) underway, a dedicated capability will be available for imaging of magnetic domains in 2D and 3D (Strempfer *et al.*, 2022 ▸). The beamline will provide a nanofocused beam in the 100 nm to 200 nm range as well as high coherent X-ray flux for ptychography experiments, which will allow us to increase the spatial resolution. Though it is desirable to compare the 3D reconstructions obtained here with those using other existing algorithms based on different approaches, this will require making such algorithms open source and/or accessible, as done here. The reconstruction algorithm pipeline presented here can be used at any beamline capable of carrying out imaging experiments and will be made available at the POLAR beamline at the upgraded Advanced Photon Source. Our data are available on request. We hope this study presents a benchmarking possibility for the reconstruction of magnetic domains in 3D that can be used to compare different data acquisition methods.

In the future, some of the computational steps are likely to be augmented or replaced by more advanced algorithms. For example, the registration of the projection images and alignment of the 3D object corresponding to the two tilt geometries could be done using machine-learning approaches which is one of our future research directions. Additionally, using physics-based prior knowledge from micro-magnetic simulations as additional input to the 3D magnetization vector reconstruction algorithm is a potential new direction for speeding up reconstructions.

## 6. Conclusions

We developed an STXM based tomographic imaging technique that allows us to probe the three components of vectorial magnetization within spatially resolved magnetic domains in a magnetic material, with a resolution limited by the beam size and the accuracy of the position corrections for errors introduced by the instrument. Single crystals of Nd_2_Fe_14_B, a material that makes the basis for strongest permanent magnets, were nano-fabricated into cylindrical samples of 2.1 µm and 5.4 µm diameter and used as a case study to develop the reconstruction workflow. Dichroic STXM projections are acquired by rotating the 3D object in a tomographic setup using *C*_L_ and *C*_R_ polarized X-rays for two sample tilt orientations. The projections are registered using a method based on tomographic consistency and the 3D reconstructed objects for the tilt orientations are aligned. A 3D magnetization vector reconstruction algorithm has been developed with the aligned STXM based projections from these two sample tilts. The different domains are clearly visible in 3D as vectors. The best estimated spatial resolution of these magnetic domains along the *x*, *y* and *z* axes is determined by an X-ray beam size of 120 nm incident on the Nd_2_Fe_14_B sample.
